# *catena*-Poly[[[aqua­[3-(3-hy­droxy­phen­yl)prop-2-enoato]samarium(III)]-bis­[μ_2_-3-(3-hy­droxy­phen­yl)prop-2-enoato]] monohydrate]

**DOI:** 10.1107/S1600536812013724

**Published:** 2012-04-04

**Authors:** Jing-ke Guo, Yi-Hang Wen

**Affiliations:** a Zhejiang Key Laboratory for Reactive Chemistry on Solid Surfaces, College of Chemistry and Life Science, Zhejiang Normal University, Jinhua, Zhejiang 321004, People’s Republic of China

## Abstract

The title Sm^III^ compound, {[Sm(C_9_H_7_O_3_)_3_(H_2_O)]·H_2_O}_*n*_, was obtained under hydrothermal conditions. Its structure is isotypic with the analogous Eu complex. The latter was reported incorrectly in space group *P*1 by Yan *et al.* [*J. Mol. Struct.* (2008), **891**, 298–304]. This was corrected by Marsh [*Acta Cryst*. B**65**, 782–783] to *P*-1. The Sm^III^ ion is nine-coordinated by O atoms from one coordinating water molecule and the remaining ones from the 3-(3-hy­droxy­phen­yl)prop-2-enoatate anions (one bidentate, two bidentate and bridging, two monodentate bridging), leading to a distorted tricapped trigonal–prismatic coordination polyhedron surrounded by solvent water mol­ecules. In the crystal, extensive intermolecular O—H⋯O hydrogen-bonding inter­actions and π–π inter­actions [centroid–centroid separation = 3.9393 (1) Å] lead to the formation of a three-dimensional supra­molecular network.

## Related literature

For the isotypic Eu structure, see: Yan *et al.* (2008 ▶) and for the corrected space-group assignment, see: Marsh (2009 ▶). For related structures, see: Niu *et al.* (2008 ▶); Tang *et al.* (2009 ▶); Wang & Feng (2010 ▶); Xue *et al.* (2007 ▶); Ye *et al.* (2005 ▶).
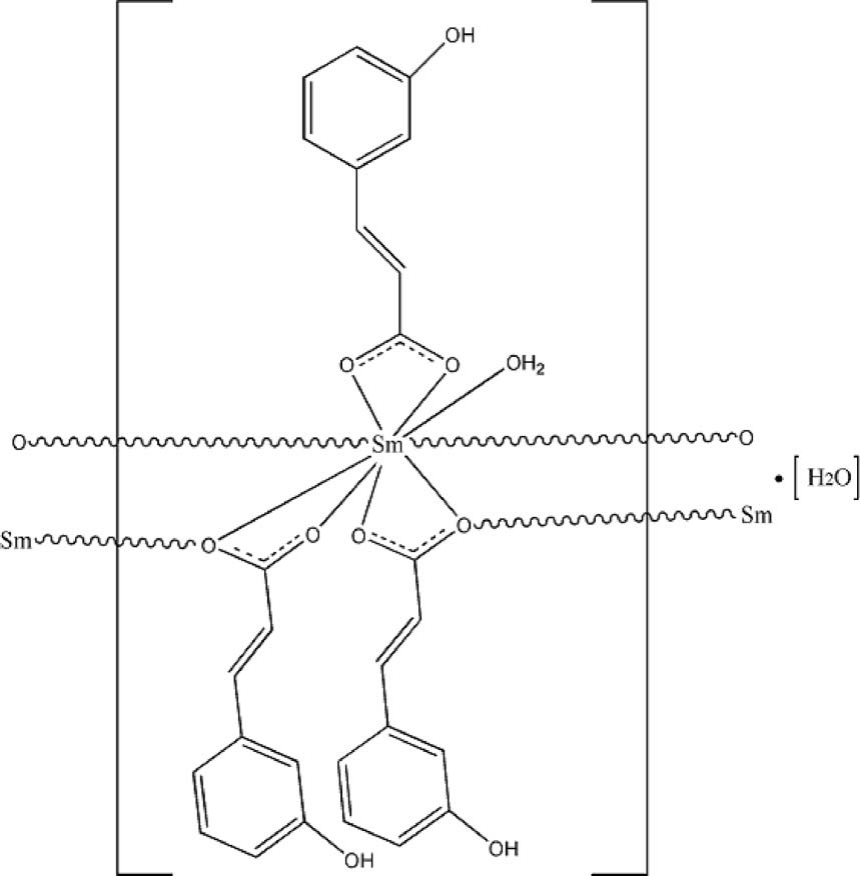


## Experimental

### 

#### Crystal data

[Sm(C_9_H_7_O_3_)_3_(H_2_O)]·H_2_O
*M* *_r_* = 675.82
Triclinic, 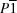

*a* = 7.9411 (1) Å
*b* = 13.0312 (2) Å
*c* = 13.6564 (2) Å
α = 97.356 (1)°
β = 97.120 (1)°
γ = 103.739 (1)°
*V* = 1343.87 (3) Å^3^
*Z* = 2
Mo *K*α radiation
μ = 2.25 mm^−1^
*T* = 296 K
0.52 × 0.24 × 0.15 mm

#### Data collection

Bruker APEXII area-detector diffractometer
Absorption correction: multi-scan (*SADABS*; Sheldrick, 1996 ▶) *T*_min_ = 0.53, *T*_max_ = 0.71
17958 measured reflections
4722 independent reflections
4590 reflections with *I* > 2σ(*I*)
*R*_int_ = 0.016

#### Refinement

*R*[*F* ^2^ > 2σ(*F* ^2^)] = 0.014
*wR*(*F* ^2^) = 0.037
*S* = 1.04
4722 reflections
373 parameters
10 restraints
H atoms treated by a mixture of independent and constrained refinement
Δρ_max_ = 0.40 e Å^−3^
Δρ_min_ = −0.47 e Å^−3^

### 

Data collection: *APEX2* (Bruker, 2006 ▶); cell refinement: *SAINT* (Bruker, 2006 ▶); data reduction: *SAINT*; program(s) used to solve structure: *SHELXS97* (Sheldrick, 2008 ▶); program(s) used to refine structure: *SHELXL97* (Sheldrick, 2008 ▶); molecular graphics: *DIAMOND* (Brandenburg, 1999 ▶); software used to prepare material for publication: *SHELXTL* (Sheldrick, 2008 ▶).

## Supplementary Material

Crystal structure: contains datablock(s) I, global. DOI: 10.1107/S1600536812013724/zj2052sup1.cif

Structure factors: contains datablock(s) I. DOI: 10.1107/S1600536812013724/zj2052Isup2.hkl

Additional supplementary materials:  crystallographic information; 3D view; checkCIF report

## Figures and Tables

**Table 1:** Hydrogen-bond geometry (Å, °)

*D*—H⋯*A*	*D*—H	H⋯*A*	*D*⋯*A*	*D*—H⋯*A*
O1*W*—H1*WA*⋯O5^i^	0.82 (2)	2.02 (2)	2.8183 (18)	163 (2)
O3—H3⋯O8^ii^	0.91 (2)	1.76 (2)	2.6692 (19)	176 (3)
O6—H6⋯O7^iii^	0.92 (2)	1.78 (2)	2.7005 (19)	174 (3)
O2*W*—H2*WA*⋯O9^iv^	0.84 (2)	2.15 (2)	2.947 (4)	158 (3)
O1*W*—H1*WB*⋯O2*W*^v^	0.83 (2)	1.86 (2)	2.688 (2)	175 (2)
O2*W*—H2*WB*⋯O3^vi^	0.81 (2)	1.98 (2)	2.790 (3)	173 (4)

Symmetry codes: (i) 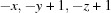; (ii) 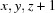; (iii) 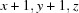; (iv) 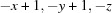; (v) 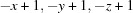; (vi) 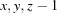.

**Fig. 1.**
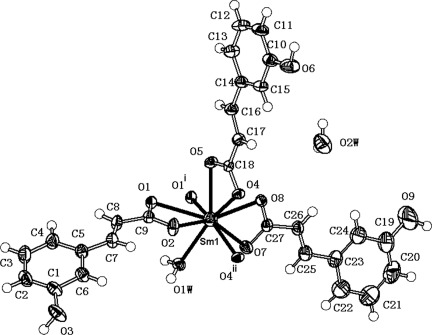
The molecular structure of title compound. Displacement ellipsoids are drawn at the 30% probability level. [Symmetry codes: (i) -x,-y + 1,-z + 1; (ii) -x + 1,-y + 1,-z + 1.]

**Fig. 2.**
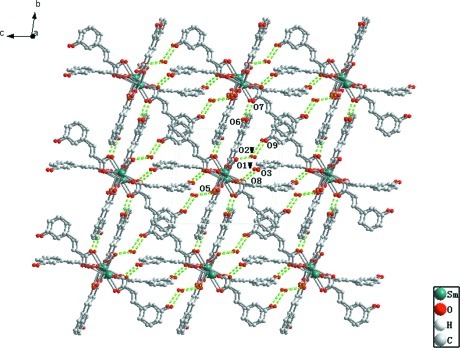
View of the supramolecular network conneted by hydrogen bonds.

## supplementary crystallographic information

### Comment

It is well known that metal and appropriate ligand are the keys for construction of metal-organic frameworks. Here we choose 3-hydroxycinnamic acid as a ligand due to its unique ability to form stable chelates in diverse coordination modes. Reserches on the compounds containing metal ions and 3-hydroxycinnamic acid have been reported (Niu *et al.* (2008); Xue *et al.*(2007); Ye *et al.*(2005)). Hererin we report a Sm^III^ compound, Sm(C_9_H_7_O_3_)_3_(H_2_O).(H_2_O), derived from 3-hydroxycinnamic acid (H_2_L),which is not the first rare-earth complex of this ligand. It is isostructural to the Eu complex, Firstly wrongly in P1 by Yan *et al.*, in 2008, this was then corrected by Marsh to P-1 in 2009.

As is shown in Fig. 1, the structrue contains only one nine-coordinated Sm^(III)^ ions which is coordinated by eight oxygen atoms from carboxylate groups in four 3-hydroxyphenyl anions and one oxygen atom from the water molecule, leading to a distored tricapped trigonal prism structure surrounded by solvent H_2_O molecule.

In the structure,the 3-hydroxycinnamic acids coordinate *via* three chelating carboxylate groups and two oxygen atoms of another two carboxylate groups bridging two Sm^(III)^ ions [Sm—O distances in the range of 2.3695 (11) and 2.5861 (11) Å], to form a one-dimensional chain with Sm···Sm 4.0887 (1) Å. Furthermore,the oxygen atom in the coordinated water molecule [Sm—O distance 2.4517 (13) Å] completes the nine-coordinated configuration of Sm. In addition,there are one lattice H_2_O molecule in the crystal structure. Intermolecular O—H···O and C—H···O hydrogen bonds connect the molecules to form a three-dimensional supramolecular skeleton (Fig. 2).

### Experimental

A mixture of Sm(NO_3_)_3_(0.1682 g, 0.5 mmol),3-hydroxycinnamic acid (0.2462 g, 1.5 mmol) and 4,4-bipyridine (0.2343 g, 1.5 mmol) was dissolved in a 16 mL EtOH/H_2_O(*v*/*v*,1:15) and then sealed in a 25 ml stainless steel reactor with a telflon liner and heated at 413 K for 72 h, and then cooled to room temperature over 3 days. Then, the reactor was cooled to room temperature at a speed of 5 degrees per hour. Yellow single crystals of title compound were obtained by slow evaporation of the filtrate over a few days.

### Refinement

The carbon-bound H-atoms were positioned geometrically and included in the refinement using a riding model [C—H 0.93 Å *U*_iso_(H) = 1.2*U*_eq_(C)]. Water H atoms were located in different maps and refined with distance restraints of O—H = 0.85 (2) Å and H—H = 1.35 Å, with displacement parameters set at 1.5*U*_eq_(O).

### Crystal data

**Table:**

[Sm(C_9_H_7_O_3_)_3_(H_2_O)]·H_2_O	*Z* = 2
*M**_r_* = 675.82	*F*(000) = 674
Triclinic, *P*1	*D*_x_ = 1.670 Mg m^−^^3^
Hall symbol: -P 1	Mo *K*α radiation, λ = 0.71073 Å
*a* = 7.9411 (1) Å	Cell parameters from 9871 reflections
*b* = 13.0312 (2) Å	θ = 1.6–25.0°
*c* = 13.6564 (2) Å	µ = 2.25 mm^−^^1^
α = 97.356 (1)°	*T* = 296 K
β = 97.120 (1)°	Block, yellow
γ = 103.739 (1)°	0.52 × 0.24 × 0.15 mm
*V* = 1343.87 (3) Å^3^	

### Data collection

**Table:**

Bruker APEXII area-detector diffractometer	4722 independent reflections
Radiation source: fine-focus sealed tube	4590 reflections with *I* > 2σ(*I*)
Graphite monochromator	*R*_int_ = 0.016
ω scans	θ_max_ = 25.0°, θ_min_ = 1.6°
Absorption correction: multi-scan (*SADABS*; Sheldrick, 1996)	*h* = −9→9
*T*_min_ = 0.53, *T*_max_ = 0.71	*k* = −15→15
17958 measured reflections	*l* = −16→16

### Refinement

**Table:**

Refinement on *F*^2^	Primary atom site location: structure-invariant direct methods
Least-squares matrix: full	Secondary atom site location: difference Fourier map
*R*[*F*^2^ > 2σ(*F*^2^)] = 0.014	Hydrogen site location: inferred from neighbouring sites
*wR*(*F*^2^) = 0.037	H atoms treated by a mixture of independent and constrained refinement
*S* = 1.04	*w* = 1/[σ^2^(*F*_o_^2^) + (0.0243*P*)^2^ + 0.3656*P*] where *P* = (*F*_o_^2^ + 2*F*_c_^2^)/3
4722 reflections	(Δ/σ)_max_ = 0.002
373 parameters	Δρ_max_ = 0.40 e Å^−^^3^
10 restraints	Δρ_min_ = −0.47 e Å^−^^3^

### Special details

**Table:**

Geometry. All e.s.d.'s (except the e.s.d. in the dihedral angle between two l.s. planes) are estimated using the full covariance matrix. The cell e.s.d.'s are taken into account individually in the estimation of e.s.d.'s in distances, angles and torsion angles; correlations between e.s.d.'s in cell parameters are only used when they are defined by crystal symmetry. An approximate (isotropic) treatment of cell e.s.d.'s is used for estimating e.s.d.'s involving l.s. planes.
Refinement. Refinement of *F*^2^ against ALL reflections. The weighted *R*-factor *wR* and goodness of fit *S* are based on *F*^2^, conventional *R*-factors *R* are based on *F*, with *F* set to zero for negative *F*^2^. The threshold expression of *F*^2^ > σ(*F*^2^) is used only for calculating *R*-factors(gt) *etc*. and is not relevant to the choice of reflections for refinement. *R*-factors based on *F*^2^ are statistically about twice as large as those based on *F*, and *R*- factors based on ALL data will be even larger.

### Fractional atomic coordinates and isotropic or equivalent isotropic displacement parameters (Å^2^)

**Table:**

	*x*	*y*	*z*	*U*_iso_*/*U*_eq_	
Sm1	0.231707 (9)	0.462033 (5)	0.480253 (5)	0.01695 (4)	
O1	0.06012 (15)	0.55519 (9)	0.59671 (8)	0.0240 (2)	
O1W	0.07151 (18)	0.32289 (11)	0.56401 (11)	0.0324 (3)	
H1WA	−0.034 (2)	0.318 (2)	0.5643 (17)	0.049*	
H1WB	0.109 (3)	0.312 (2)	0.6205 (14)	0.049*	
O2	0.32583 (16)	0.54888 (12)	0.65526 (9)	0.0349 (3)	
O2W	0.7925 (2)	0.70266 (18)	0.25319 (14)	0.0629 (5)	
H2WA	0.786 (4)	0.7600 (17)	0.232 (3)	0.094*	
H2WB	0.697 (3)	0.661 (2)	0.232 (3)	0.094*	
O3	0.4554 (2)	0.56809 (15)	1.19165 (12)	0.0515 (4)	
H3	0.401 (3)	0.5246 (19)	1.2314 (18)	0.062*	
O4	0.51185 (15)	0.59885 (9)	0.47391 (9)	0.0240 (3)	
O5	0.26831 (15)	0.64608 (9)	0.43899 (10)	0.0278 (3)	
O6	1.0331 (2)	1.09500 (12)	0.39523 (14)	0.0506 (4)	
H6	1.080 (3)	1.1627 (15)	0.382 (2)	0.061*	
O7	0.19076 (17)	0.29241 (9)	0.36232 (9)	0.0306 (3)	
O8	0.28389 (16)	0.44152 (9)	0.30584 (9)	0.0259 (3)	
O9	0.3333 (5)	0.1288 (2)	−0.16532 (17)	0.1181 (11)	
H9	0.351 (6)	0.077 (3)	−0.215 (3)	0.142*	
C1	0.3318 (3)	0.59470 (16)	1.12695 (14)	0.0358 (4)	
C2	0.1850 (3)	0.62021 (18)	1.15591 (15)	0.0437 (5)	
H2A	0.1672	0.6218	1.2221	0.052*	
C3	0.0638 (3)	0.6435 (2)	1.08592 (16)	0.0479 (6)	
H3A	−0.0346	0.6618	1.1056	0.057*	
C4	0.0877 (3)	0.63986 (18)	0.98720 (15)	0.0401 (5)	
H4A	0.0039	0.6541	0.9406	0.048*	
C5	0.2359 (3)	0.61510 (15)	0.95721 (14)	0.0313 (4)	
C6	0.3595 (3)	0.59493 (16)	1.02878 (14)	0.0349 (4)	
H6A	0.4621	0.5814	1.0104	0.042*	
C7	0.2629 (2)	0.60020 (16)	0.85277 (14)	0.0320 (4)	
H7A	0.3726	0.5922	0.8417	0.038*	
C8	0.1498 (2)	0.59684 (16)	0.77289 (13)	0.0306 (4)	
H8A	0.0447	0.6140	0.7811	0.037*	
C9	0.1830 (2)	0.56691 (14)	0.67091 (13)	0.0237 (4)	
C10	0.8553 (3)	1.07639 (15)	0.38269 (15)	0.0351 (4)	
C11	0.7683 (3)	1.14997 (15)	0.35253 (16)	0.0406 (5)	
H11A	0.8315	1.2160	0.3411	0.049*	
C12	0.5879 (3)	1.12497 (16)	0.33946 (18)	0.0457 (5)	
H12A	0.5299	1.1749	0.3196	0.055*	
C13	0.4906 (3)	1.02652 (16)	0.35534 (17)	0.0395 (5)	
H13A	0.3686	1.0104	0.3454	0.047*	
C14	0.5770 (3)	0.95222 (14)	0.38628 (14)	0.0305 (4)	
C15	0.7603 (3)	0.97840 (14)	0.40027 (15)	0.0329 (4)	
H15A	0.8194	0.9296	0.4216	0.040*	
C16	0.4745 (3)	0.84864 (14)	0.40343 (14)	0.0306 (4)	
H16A	0.3529	0.8359	0.3920	0.037*	
C17	0.5399 (2)	0.77163 (14)	0.43369 (14)	0.0291 (4)	
H17A	0.6614	0.7836	0.4458	0.035*	
C18	0.4330 (2)	0.66875 (13)	0.44924 (12)	0.0214 (3)	
C19	0.2960 (5)	0.0830 (2)	−0.0827 (2)	0.0676 (8)	
C20	0.2780 (5)	−0.0240 (2)	−0.0819 (2)	0.0729 (9)	
H20A	0.2905	−0.0681	−0.1383	0.087*	
C21	0.2415 (5)	−0.0653 (2)	0.0023 (3)	0.0786 (10)	
H21A	0.2308	−0.1377	0.0036	0.094*	
C22	0.2202 (4)	−0.0002 (2)	0.0858 (2)	0.0643 (8)	
H22A	0.1926	−0.0294	0.1422	0.077*	
C23	0.2398 (3)	0.10820 (18)	0.08566 (17)	0.0436 (5)	
C24	0.2779 (4)	0.1496 (2)	0.00018 (18)	0.0572 (7)	
H24A	0.2912	0.2222	−0.0014	0.069*	
C25	0.2225 (3)	0.17526 (16)	0.17687 (15)	0.0390 (5)	
H25A	0.1776	0.1391	0.2262	0.047*	
C26	0.2632 (3)	0.28090 (15)	0.19707 (14)	0.0319 (4)	
H26A	0.3052	0.3196	0.1486	0.038*	
C27	0.2452 (2)	0.34016 (14)	0.29289 (13)	0.0241 (4)	

### Atomic displacement parameters (Å^2^)

**Table:**

	*U*^11^	*U*^22^	*U*^33^	*U*^12^	*U*^13^	*U*^23^
Sm1	0.01437 (6)	0.01882 (5)	0.01808 (6)	0.00429 (3)	0.00325 (3)	0.00391 (3)
O1	0.0194 (6)	0.0320 (6)	0.0198 (6)	0.0078 (5)	0.0009 (5)	0.0018 (5)
O1W	0.0274 (7)	0.0365 (7)	0.0393 (8)	0.0103 (6)	0.0122 (6)	0.0173 (6)
O2	0.0209 (6)	0.0605 (9)	0.0234 (6)	0.0171 (6)	0.0007 (5)	−0.0023 (6)
O2W	0.0519 (11)	0.0872 (14)	0.0500 (11)	0.0134 (10)	0.0081 (8)	0.0216 (10)
O3	0.0422 (9)	0.0771 (11)	0.0373 (8)	0.0086 (8)	0.0032 (7)	0.0328 (8)
O4	0.0185 (6)	0.0208 (6)	0.0340 (7)	0.0063 (5)	0.0027 (5)	0.0084 (5)
O5	0.0190 (6)	0.0254 (6)	0.0420 (7)	0.0076 (5)	0.0066 (5)	0.0109 (5)
O6	0.0413 (9)	0.0343 (8)	0.0739 (12)	−0.0010 (7)	0.0068 (8)	0.0225 (8)
O7	0.0377 (7)	0.0244 (6)	0.0275 (7)	0.0027 (5)	0.0086 (6)	0.0033 (5)
O8	0.0275 (6)	0.0257 (6)	0.0250 (6)	0.0059 (5)	0.0077 (5)	0.0046 (5)
O9	0.224 (3)	0.0984 (18)	0.0477 (12)	0.057 (2)	0.0579 (17)	0.0054 (12)
C1	0.0355 (11)	0.0413 (11)	0.0270 (10)	0.0019 (8)	0.0010 (8)	0.0113 (8)
C2	0.0549 (14)	0.0517 (13)	0.0255 (10)	0.0117 (10)	0.0127 (10)	0.0080 (9)
C3	0.0507 (14)	0.0636 (15)	0.0366 (12)	0.0248 (11)	0.0167 (10)	0.0062 (10)
C4	0.0420 (12)	0.0539 (12)	0.0298 (10)	0.0225 (10)	0.0066 (9)	0.0067 (9)
C5	0.0336 (10)	0.0350 (10)	0.0244 (9)	0.0083 (8)	0.0038 (8)	0.0033 (7)
C6	0.0297 (10)	0.0445 (11)	0.0302 (10)	0.0073 (8)	0.0038 (8)	0.0095 (8)
C7	0.0271 (9)	0.0444 (11)	0.0255 (9)	0.0113 (8)	0.0050 (8)	0.0039 (8)
C8	0.0260 (9)	0.0455 (11)	0.0231 (9)	0.0159 (8)	0.0047 (8)	0.0022 (8)
C9	0.0225 (9)	0.0256 (8)	0.0217 (9)	0.0056 (7)	0.0024 (7)	0.0017 (7)
C10	0.0425 (12)	0.0257 (9)	0.0333 (10)	0.0021 (8)	0.0039 (9)	0.0059 (8)
C11	0.0544 (13)	0.0226 (9)	0.0425 (12)	0.0029 (9)	0.0067 (10)	0.0115 (8)
C12	0.0588 (15)	0.0289 (10)	0.0549 (14)	0.0175 (10)	0.0061 (11)	0.0179 (10)
C13	0.0418 (12)	0.0326 (10)	0.0466 (12)	0.0125 (9)	0.0050 (10)	0.0121 (9)
C14	0.0398 (11)	0.0236 (9)	0.0287 (9)	0.0075 (8)	0.0070 (8)	0.0061 (7)
C15	0.0391 (11)	0.0235 (9)	0.0373 (10)	0.0069 (8)	0.0053 (9)	0.0116 (8)
C16	0.0304 (10)	0.0258 (9)	0.0370 (10)	0.0069 (7)	0.0072 (8)	0.0089 (8)
C17	0.0239 (9)	0.0256 (9)	0.0389 (10)	0.0052 (7)	0.0087 (8)	0.0086 (8)
C18	0.0215 (9)	0.0216 (8)	0.0222 (8)	0.0068 (6)	0.0047 (7)	0.0036 (6)
C19	0.096 (2)	0.0696 (18)	0.0382 (14)	0.0266 (16)	0.0186 (14)	−0.0042 (12)
C20	0.095 (2)	0.0618 (18)	0.0569 (18)	0.0250 (16)	0.0176 (16)	−0.0221 (14)
C21	0.116 (3)	0.0413 (14)	0.074 (2)	0.0186 (15)	0.0223 (19)	−0.0111 (14)
C22	0.091 (2)	0.0405 (13)	0.0580 (16)	0.0123 (13)	0.0192 (15)	−0.0024 (12)
C23	0.0502 (13)	0.0402 (12)	0.0371 (12)	0.0117 (10)	0.0068 (10)	−0.0057 (9)
C24	0.086 (2)	0.0462 (13)	0.0402 (13)	0.0215 (13)	0.0153 (13)	−0.0018 (10)
C25	0.0472 (12)	0.0391 (11)	0.0306 (10)	0.0121 (9)	0.0084 (9)	0.0021 (8)
C26	0.0350 (10)	0.0364 (10)	0.0251 (9)	0.0112 (8)	0.0072 (8)	0.0019 (8)
C27	0.0175 (8)	0.0307 (9)	0.0240 (9)	0.0070 (7)	0.0024 (7)	0.0030 (7)

### Geometric parameters (Å, º)

**Table:**

Sm1—O1^i^	2.3695 (11)	C5—C6	1.392 (3)
Sm1—O4^ii^	2.3973 (11)	C5—C7	1.464 (3)
Sm1—O1W	2.4517 (13)	C6—H6A	0.9300
Sm1—O2	2.4524 (12)	C7—C8	1.313 (3)
Sm1—O8	2.4598 (12)	C7—H7A	0.9300
Sm1—O7	2.4905 (12)	C8—C9	1.470 (2)
Sm1—O5	2.4919 (12)	C8—H8A	0.9300
Sm1—O4	2.5176 (11)	C10—C11	1.382 (3)
Sm1—O1	2.5861 (11)	C10—C15	1.387 (3)
Sm1—C27	2.8616 (17)	C11—C12	1.375 (3)
Sm1—C9	2.8959 (17)	C11—H11A	0.9300
Sm1—C18	2.8991 (16)	C12—C13	1.390 (3)
O1—C9	1.283 (2)	C12—H12A	0.9300
O1—Sm1^i^	2.3695 (11)	C13—C14	1.391 (3)
O1W—H1WA	0.824 (15)	C13—H13A	0.9300
O1W—H1WB	0.834 (16)	C14—C15	1.397 (3)
O2—C9	1.249 (2)	C14—C16	1.465 (2)
O2W—H2WA	0.844 (17)	C15—H15A	0.9300
O2W—H2WB	0.814 (17)	C16—C17	1.322 (3)
O3—C1	1.374 (3)	C16—H16A	0.9300
O3—H3	0.906 (17)	C17—C18	1.463 (2)
O4—C18	1.278 (2)	C17—H17A	0.9300
O4—Sm1^ii^	2.3973 (11)	C19—C20	1.369 (4)
O5—C18	1.256 (2)	C19—C24	1.379 (3)
O6—C10	1.360 (3)	C20—C21	1.365 (5)
O6—H6	0.923 (17)	C20—H20A	0.9300
O7—C27	1.264 (2)	C21—C22	1.384 (4)
O8—C27	1.267 (2)	C21—H21A	0.9300
O9—C19	1.374 (4)	C22—C23	1.384 (3)
O9—H9	0.944 (19)	C22—H22A	0.9300
C1—C2	1.375 (3)	C23—C24	1.384 (4)
C1—C6	1.385 (3)	C23—C25	1.468 (3)
C2—C3	1.385 (3)	C24—H24A	0.9300
C2—H2A	0.9300	C25—C26	1.321 (3)
C3—C4	1.381 (3)	C25—H25A	0.9300
C3—H3A	0.9300	C26—C27	1.473 (2)
C4—C5	1.387 (3)	C26—H26A	0.9300
C4—H4A	0.9300		
			
O1^i^—Sm1—O4^ii^	155.55 (4)	C1—C2—H2A	120.2
O1^i^—Sm1—O1W	79.70 (5)	C3—C2—H2A	120.2
O4^ii^—Sm1—O1W	87.34 (4)	C4—C3—C2	120.7 (2)
O1^i^—Sm1—O2	119.15 (4)	C4—C3—H3A	119.7
O4^ii^—Sm1—O2	78.18 (4)	C2—C3—H3A	119.7
O1W—Sm1—O2	79.90 (5)	C3—C4—C5	120.3 (2)
O1^i^—Sm1—O8	82.67 (4)	C3—C4—H4A	119.9
O4^ii^—Sm1—O8	88.82 (4)	C5—C4—H4A	119.9
O1W—Sm1—O8	126.86 (4)	C4—C5—C6	118.54 (18)
O2—Sm1—O8	150.00 (4)	C4—C5—C7	123.46 (18)
O1^i^—Sm1—O7	81.62 (4)	C6—C5—C7	117.80 (17)
O4^ii^—Sm1—O7	75.09 (4)	C1—C6—C5	120.92 (19)
O1W—Sm1—O7	75.65 (4)	C1—C6—H6A	119.5
O2—Sm1—O7	144.28 (5)	C5—C6—H6A	119.5
O8—Sm1—O7	52.30 (4)	C8—C7—C5	127.42 (18)
O1^i^—Sm1—O5	81.25 (4)	C8—C7—H7A	116.3
O4^ii^—Sm1—O5	119.02 (4)	C5—C7—H7A	116.3
O1W—Sm1—O5	147.03 (4)	C7—C8—C9	122.50 (17)
O2—Sm1—O5	86.30 (5)	C7—C8—H8A	118.8
O8—Sm1—O5	76.50 (4)	C9—C8—H8A	118.8
O7—Sm1—O5	127.52 (4)	O2—C9—O1	119.34 (15)
O1^i^—Sm1—O4	130.35 (4)	O2—C9—C8	121.45 (16)
O4^ii^—Sm1—O4	67.43 (4)	O1—C9—C8	119.16 (15)
O1W—Sm1—O4	148.72 (4)	O2—C9—Sm1	57.08 (9)
O2—Sm1—O4	77.13 (4)	O1—C9—Sm1	63.25 (8)
O8—Sm1—O4	72.92 (4)	C8—C9—Sm1	167.72 (13)
O7—Sm1—O4	112.86 (4)	O6—C10—C11	122.73 (17)
O5—Sm1—O4	51.62 (4)	O6—C10—C15	117.38 (19)
O1^i^—Sm1—O1	67.98 (4)	C11—C10—C15	119.9 (2)
O4^ii^—Sm1—O1	128.01 (4)	C12—C11—C10	119.62 (18)
O1W—Sm1—O1	73.82 (4)	C12—C11—H11A	120.2
O2—Sm1—O1	51.34 (4)	C10—C11—H11A	120.2
O8—Sm1—O1	140.98 (4)	C11—C12—C13	121.2 (2)
O7—Sm1—O1	139.96 (4)	C11—C12—H12A	119.4
O5—Sm1—O1	74.17 (4)	C13—C12—H12A	119.4
O4—Sm1—O1	106.71 (4)	C12—C13—C14	119.5 (2)
O1^i^—Sm1—C27	82.20 (4)	C12—C13—H13A	120.2
O4^ii^—Sm1—C27	80.18 (5)	C14—C13—H13A	120.2
O1W—Sm1—C27	101.49 (5)	C13—C14—C15	118.97 (18)
O2—Sm1—C27	158.23 (5)	C13—C14—C16	119.57 (18)
O8—Sm1—C27	26.17 (5)	C15—C14—C16	121.46 (17)
O7—Sm1—C27	26.16 (5)	C10—C15—C14	120.72 (19)
O5—Sm1—C27	102.28 (5)	C10—C15—H15A	119.6
O4—Sm1—C27	92.42 (4)	C14—C15—H15A	119.6
O1—Sm1—C27	150.18 (4)	C17—C16—C14	125.68 (18)
O1^i^—Sm1—C9	93.84 (4)	C17—C16—H16A	117.2
O4^ii^—Sm1—C9	102.04 (5)	C14—C16—H16A	117.2
O1W—Sm1—C9	72.73 (5)	C16—C17—C18	124.04 (17)
O2—Sm1—C9	25.32 (5)	C16—C17—H17A	118.0
O8—Sm1—C9	158.51 (5)	C18—C17—H17A	118.0
O7—Sm1—C9	148.35 (4)	O5—C18—O4	118.84 (15)
O5—Sm1—C9	82.02 (5)	O5—C18—C17	123.16 (16)
O4—Sm1—C9	94.00 (4)	O4—C18—C17	118.00 (15)
O1—Sm1—C9	26.30 (4)	O5—C18—Sm1	58.79 (8)
C27—Sm1—C9	173.57 (5)	O4—C18—Sm1	60.05 (8)
O1^i^—Sm1—C18	105.70 (4)	C17—C18—Sm1	178.01 (12)
O4^ii^—Sm1—C18	93.51 (4)	C20—C19—O9	121.7 (2)
O1W—Sm1—C18	160.17 (5)	C20—C19—C24	121.1 (3)
O2—Sm1—C18	80.89 (5)	O9—C19—C24	117.2 (3)
O8—Sm1—C18	72.97 (4)	C21—C20—C19	119.3 (2)
O7—Sm1—C18	123.71 (4)	C21—C20—H20A	120.3
O5—Sm1—C18	25.53 (4)	C19—C20—H20A	120.3
O4—Sm1—C18	26.09 (4)	C20—C21—C22	120.5 (3)
O1—Sm1—C18	90.30 (4)	C20—C21—H21A	119.7
C27—Sm1—C18	98.18 (5)	C22—C21—H21A	119.7
C9—Sm1—C18	87.75 (5)	C23—C22—C21	120.3 (3)
C9—O1—Sm1^i^	155.04 (11)	C23—C22—H22A	119.9
C9—O1—Sm1	90.44 (9)	C21—C22—H22A	119.9
Sm1^i^—O1—Sm1	112.02 (4)	C22—C23—C24	118.8 (2)
Sm1—O1W—H1WA	117.4 (17)	C22—C23—C25	118.7 (2)
Sm1—O1W—H1WB	123.4 (17)	C24—C23—C25	122.5 (2)
H1WA—O1W—H1WB	104.8 (19)	C19—C24—C23	119.9 (3)
C9—O2—Sm1	97.61 (10)	C19—C24—H24A	120.0
H2WA—O2W—H2WB	105 (2)	C23—C24—H24A	120.0
C1—O3—H3	109.8 (18)	C26—C25—C23	127.6 (2)
C18—O4—Sm1^ii^	153.49 (10)	C26—C25—H25A	116.2
C18—O4—Sm1	93.86 (9)	C23—C25—H25A	116.2
Sm1^ii^—O4—Sm1	112.57 (4)	C25—C26—C27	123.00 (19)
C18—O5—Sm1	95.67 (10)	C25—C26—H26A	118.5
C10—O6—H6	108.7 (17)	C27—C26—H26A	118.5
C27—O7—Sm1	93.54 (10)	O7—C27—O8	119.10 (15)
C27—O8—Sm1	94.92 (10)	O7—C27—C26	121.68 (16)
C19—O9—H9	109 (3)	O8—C27—C26	119.22 (16)
O3—C1—C2	122.80 (18)	O7—C27—Sm1	60.30 (9)
O3—C1—C6	117.30 (19)	O8—C27—Sm1	58.92 (8)
C2—C1—C6	119.90 (19)	C26—C27—Sm1	176.60 (13)
C1—C2—C3	119.56 (19)		
			
O1^i^—Sm1—O1—C9	168.85 (12)	O1^i^—Sm1—C9—O2	−178.87 (11)
O4^ii^—Sm1—O1—C9	10.38 (11)	O4^ii^—Sm1—C9—O2	19.83 (12)
O1W—Sm1—O1—C9	83.64 (10)	O1W—Sm1—C9—O2	103.17 (12)
O2—Sm1—O1—C9	−6.26 (10)	O8—Sm1—C9—O2	−99.16 (16)
O8—Sm1—O1—C9	−147.01 (10)	O7—Sm1—C9—O2	100.89 (13)
O7—Sm1—O1—C9	125.36 (10)	O5—Sm1—C9—O2	−98.27 (11)
O5—Sm1—O1—C9	−104.36 (10)	O4—Sm1—C9—O2	−47.93 (12)
O4—Sm1—O1—C9	−63.72 (10)	O1—Sm1—C9—O2	−168.52 (18)
C27—Sm1—O1—C9	168.52 (10)	C18—Sm1—C9—O2	−73.28 (12)
C18—Sm1—O1—C9	−84.31 (10)	O1^i^—Sm1—C9—O1	−10.35 (11)
O1^i^—Sm1—O1—Sm1^i^	0.0	O4^ii^—Sm1—C9—O1	−171.66 (9)
O4^ii^—Sm1—O1—Sm1^i^	−158.47 (4)	O1W—Sm1—C9—O1	−88.32 (10)
O1W—Sm1—O1—Sm1^i^	−85.21 (6)	O2—Sm1—C9—O1	168.52 (17)
O2—Sm1—O1—Sm1^i^	−175.11 (8)	O8—Sm1—C9—O1	69.36 (16)
O8—Sm1—O1—Sm1^i^	44.14 (8)	O7—Sm1—C9—O1	−90.60 (13)
O7—Sm1—O1—Sm1^i^	−43.49 (8)	O5—Sm1—C9—O1	70.24 (9)
O5—Sm1—O1—Sm1^i^	86.80 (5)	O4—Sm1—C9—O1	120.58 (9)
O4—Sm1—O1—Sm1^i^	127.43 (5)	C18—Sm1—C9—O1	95.23 (10)
C27—Sm1—O1—Sm1^i^	−0.32 (11)	O1^i^—Sm1—C9—C8	94.1 (6)
C9—Sm1—O1—Sm1^i^	−168.85 (12)	O4^ii^—Sm1—C9—C8	−67.2 (6)
C18—Sm1—O1—Sm1^i^	106.84 (5)	O1W—Sm1—C9—C8	16.2 (6)
O1^i^—Sm1—O2—C9	1.29 (13)	O2—Sm1—C9—C8	−87.0 (6)
O4^ii^—Sm1—O2—C9	−160.19 (12)	O8—Sm1—C9—C8	173.9 (5)
O1W—Sm1—O2—C9	−70.81 (11)	O7—Sm1—C9—C8	13.9 (6)
O8—Sm1—O2—C9	133.68 (11)	O5—Sm1—C9—C8	174.7 (6)
O7—Sm1—O2—C9	−118.04 (12)	O4—Sm1—C9—C8	−134.9 (6)
O5—Sm1—O2—C9	79.13 (11)	O1—Sm1—C9—C8	104.5 (6)
O4—Sm1—O2—C9	130.57 (12)	C18—Sm1—C9—C8	−160.3 (6)
O1—Sm1—O2—C9	6.49 (10)	O6—C10—C11—C12	178.7 (2)
C27—Sm1—O2—C9	−166.51 (12)	C15—C10—C11—C12	−0.5 (3)
C18—Sm1—O2—C9	104.25 (12)	C10—C11—C12—C13	−0.5 (3)
O1^i^—Sm1—O4—C18	21.91 (11)	C11—C12—C13—C14	0.8 (3)
O4^ii^—Sm1—O4—C18	−177.95 (12)	C12—C13—C14—C15	−0.3 (3)
O1W—Sm1—O4—C18	−139.30 (10)	C12—C13—C14—C16	179.75 (19)
O2—Sm1—O4—C18	−95.58 (10)	O6—C10—C15—C14	−178.21 (18)
O8—Sm1—O4—C18	86.05 (10)	C11—C10—C15—C14	1.1 (3)
O7—Sm1—O4—C18	120.58 (9)	C13—C14—C15—C10	−0.7 (3)
O5—Sm1—O4—C18	−0.04 (9)	C16—C14—C15—C10	179.31 (18)
O1—Sm1—O4—C18	−53.10 (10)	C13—C14—C16—C17	−179.3 (2)
C27—Sm1—O4—C18	103.73 (10)	C15—C14—C16—C17	0.7 (3)
C9—Sm1—O4—C18	−76.57 (10)	C14—C16—C17—C18	−179.51 (17)
O1^i^—Sm1—O4—Sm1^ii^	−160.13 (4)	Sm1—O5—C18—O4	−0.08 (16)
O4^ii^—Sm1—O4—Sm1^ii^	0.0	Sm1—O5—C18—C17	179.55 (15)
O1W—Sm1—O4—Sm1^ii^	38.66 (10)	Sm1^ii^—O4—C18—O5	−175.69 (16)
O2—Sm1—O4—Sm1^ii^	82.38 (5)	Sm1—O4—C18—O5	0.08 (16)
O8—Sm1—O4—Sm1^ii^	−96.00 (5)	Sm1^ii^—O4—C18—C17	4.7 (3)
O7—Sm1—O4—Sm1^ii^	−61.47 (6)	Sm1—O4—C18—C17	−179.57 (13)
O5—Sm1—O4—Sm1^ii^	177.91 (7)	Sm1^ii^—O4—C18—Sm1	−175.8 (3)
O1—Sm1—O4—Sm1^ii^	124.86 (5)	C16—C17—C18—O5	−3.0 (3)
C27—Sm1—O4—Sm1^ii^	−78.32 (6)	C16—C17—C18—O4	176.63 (17)
C9—Sm1—O4—Sm1^ii^	101.38 (6)	O1^i^—Sm1—C18—O5	17.26 (11)
C18—Sm1—O4—Sm1^ii^	177.95 (12)	O4^ii^—Sm1—C18—O5	−178.03 (10)
O1^i^—Sm1—O5—C18	−163.20 (10)	O1W—Sm1—C18—O5	−86.14 (17)
O4^ii^—Sm1—O5—C18	2.25 (11)	O2—Sm1—C18—O5	−100.60 (10)
O1W—Sm1—O5—C18	141.53 (11)	O8—Sm1—C18—O5	94.28 (10)
O2—Sm1—O5—C18	76.54 (10)	O7—Sm1—C18—O5	107.58 (10)
O8—Sm1—O5—C18	−78.70 (10)	O4—Sm1—C18—O5	−179.92 (16)
O7—Sm1—O5—C18	−90.91 (11)	O1—Sm1—C18—O5	−49.91 (10)
O4—Sm1—O5—C18	0.04 (9)	C27—Sm1—C18—O5	101.40 (10)
O1—Sm1—O5—C18	127.32 (10)	C9—Sm1—C18—O5	−76.10 (10)
C27—Sm1—O5—C18	−83.23 (10)	O1^i^—Sm1—C18—O4	−162.82 (9)
C9—Sm1—O5—C18	101.64 (10)	O4^ii^—Sm1—C18—O4	1.89 (11)
O1^i^—Sm1—O7—C27	89.35 (11)	O1W—Sm1—C18—O4	93.79 (16)
O4^ii^—Sm1—O7—C27	−98.15 (11)	O2—Sm1—C18—O4	79.32 (9)
O1W—Sm1—O7—C27	170.79 (11)	O8—Sm1—C18—O4	−85.80 (9)
O2—Sm1—O7—C27	−140.97 (10)	O7—Sm1—C18—O4	−72.50 (10)
O8—Sm1—O7—C27	2.16 (9)	O5—Sm1—C18—O4	179.92 (16)
O5—Sm1—O7—C27	17.23 (12)	O1—Sm1—C18—O4	130.01 (9)
O4—Sm1—O7—C27	−41.05 (11)	C27—Sm1—C18—O4	−78.68 (10)
O1—Sm1—O7—C27	129.50 (10)	C9—Sm1—C18—O4	103.82 (10)
C9—Sm1—O7—C27	173.03 (10)	C24—C19—C20—C21	0.0 (6)
C18—Sm1—O7—C27	−13.98 (12)	C19—C20—C21—C22	0.9 (6)
O1^i^—Sm1—O8—C27	−87.21 (10)	C20—C21—C22—C23	−1.5 (6)
O4^ii^—Sm1—O8—C27	69.81 (10)	C21—C22—C23—C24	1.1 (5)
O1W—Sm1—O8—C27	−15.98 (12)	C21—C22—C23—C25	−177.8 (3)
O2—Sm1—O8—C27	133.36 (11)	C20—C19—C24—C23	−0.4 (5)
O7—Sm1—O8—C27	−2.16 (9)	O9—C19—C24—C23	179.8 (3)
O5—Sm1—O8—C27	−169.92 (10)	C22—C23—C24—C19	−0.2 (5)
O4—Sm1—O8—C27	136.53 (10)	C25—C23—C24—C19	178.7 (3)
O1—Sm1—O8—C27	−127.82 (10)	C22—C23—C25—C26	168.4 (3)
C9—Sm1—O8—C27	−169.02 (11)	C24—C23—C25—C26	−10.5 (4)
C18—Sm1—O8—C27	163.84 (11)	C23—C25—C26—C27	−178.1 (2)
O3—C1—C2—C3	−178.3 (2)	Sm1—O7—C27—O8	−3.80 (17)
C6—C1—C2—C3	1.5 (3)	Sm1—O7—C27—C26	176.78 (15)
C1—C2—C3—C4	0.9 (4)	Sm1—O8—C27—O7	3.86 (17)
C2—C3—C4—C5	−1.5 (4)	Sm1—O8—C27—C26	−176.71 (14)
C3—C4—C5—C6	−0.4 (3)	C25—C26—C27—O7	0.8 (3)
C3—C4—C5—C7	174.3 (2)	C25—C26—C27—O8	−178.66 (19)
O3—C1—C6—C5	176.38 (18)	O1^i^—Sm1—C27—O7	−86.87 (10)
C2—C1—C6—C5	−3.5 (3)	O4^ii^—Sm1—C27—O7	76.12 (10)
C4—C5—C6—C1	2.9 (3)	O1W—Sm1—C27—O7	−9.11 (11)
C7—C5—C6—C1	−172.11 (18)	O2—Sm1—C27—O7	82.40 (16)
C4—C5—C7—C8	−7.3 (3)	O8—Sm1—C27—O7	−176.12 (17)
C6—C5—C7—C8	167.4 (2)	O5—Sm1—C27—O7	−166.09 (10)
C5—C7—C8—C9	−171.47 (18)	O4—Sm1—C27—O7	142.72 (10)
Sm1—O2—C9—O1	−11.77 (18)	O1—Sm1—C27—O7	−86.56 (14)
Sm1—O2—C9—C8	165.59 (15)	C18—Sm1—C27—O7	168.29 (10)
Sm1^i^—O1—C9—O2	165.90 (18)	O1^i^—Sm1—C27—O8	89.25 (10)
Sm1—O1—C9—O2	11.05 (17)	O4^ii^—Sm1—C27—O8	−107.76 (10)
Sm1^i^—O1—C9—C8	−11.5 (4)	O1W—Sm1—C27—O8	167.01 (10)
Sm1—O1—C9—C8	−166.37 (15)	O2—Sm1—C27—O8	−101.48 (16)
Sm1^i^—O1—C9—Sm1	154.8 (3)	O7—Sm1—C27—O8	176.12 (17)
C7—C8—C9—O2	−4.1 (3)	O5—Sm1—C27—O8	10.03 (10)
C7—C8—C9—O1	173.30 (18)	O4—Sm1—C27—O8	−41.16 (10)
C7—C8—C9—Sm1	75.2 (6)	O1—Sm1—C27—O8	89.55 (12)

Symmetry codes: (i) −*x*, −*y*+1, −*z*+1; (ii) −*x*+1, −*y*+1, −*z*+1.

### Hydrogen-bond geometry (Å, º)

**Table:**

*D*—H···*A*	*D*—H	H···*A*	*D*···*A*	*D*—H···*A*
O1*W*—H1*WA*···O5^i^	0.82 (2)	2.02 (2)	2.8183 (18)	163 (2)
O3—H3···O8^iii^	0.91 (2)	1.76 (2)	2.6692 (19)	176 (3)
O6—H6···O7^iv^	0.92 (2)	1.78 (2)	2.7005 (19)	174 (3)
O2*W*—H2*WA*···O9^v^	0.84 (2)	2.15 (2)	2.947 (4)	158 (3)
O1*W*—H1*WB*···O2*W*^ii^	0.83 (2)	1.86 (2)	2.688 (2)	175 (2)
O2*W*—H2*WB*···O3^vi^	0.81 (2)	1.98 (2)	2.790 (3)	173 (4)

Symmetry codes: (i) −*x*, −*y*+1, −*z*+1; (ii) −*x*+1, −*y*+1, −*z*+1; (iii) *x*, *y*, *z*+1; (iv) *x*+1, *y*+1, *z*; (v) −*x*+1, −*y*+1, −*z*; (vi) *x*, *y*, *z*−1.
